# Consul-General Worman’s Correspondence

**Published:** 1903-02

**Authors:** 


### Foreign Correspondence.

### CONSUL-GENERAL WORMAN'S CORRESPONDENCE.

United States Consulate-General,
Munich, Bavaria, December 18, 1902.

To the Editor:

Sir,—Professor Miller has just sent me his copy of the report of the Proceedings of the National Association of Dental Faculties, and I learn therefrom, on page 113, that the evidence against Dr. J. H. Smyser, in connection with the Finley forgery and the Igney illegal sale of license, was obtained by the prosecuting committee. This is a mistake and should be duly corrected. That information was given by me, and I myself obtained that evidence before leaving Milwaukee, by careful study of the minutes of the Board of Dental Examiners of Illinois in the case of Finley, and put it in the form of an affidavit before United States Senator Quarles at Milwaukee before proceeding to Chicago, and before such prosecuting committee had been recognized or even appointed.

Secondly, the information concerning Igney was obtained by me by anonymous correspondence while I was engaged in my investigation in Chicago, after the meeting at Milwaukee, and turned over by me to the attorney of the prosecuting committee, Mr. Knickerbocker, with whom this case was worked up before I left the city of Chicago.

As these two cases are the most positive evidence ever furnished against said Smyser, and as upon the strength of this evidence I caused the removal of the old Board of Dental Examiners by the governor, it is but fair that the profession should see to it that due correction is promptly made.

Sincerely yours,

J. H. Worman,

*United States Consul-General.*

United States Consulate-General,
Munich, Bavaria, September 25, 1902.

Dr. J. N. Crouse,

*Chairman of the Prosecuting Committee,*

Chicago, Ill.

Dear Sir,—The Huxmannites of Germany have formed a Union, and their presiding officers have informed the courts officially and under oath that the sum of $2950 raised at my urgent solicitation by the National Association of Dental Faculties at its annual meeting in 1901, and the $1000 raised by the National Dental Association for the employment of counsel and the purpose of investigating the affairs of the Illinois Board of Dental Examiners, as well as the conduct of certain colleges in issuing degrees illegally, were sums paid over to me fcr services against Huxmann, and that my sworn testimony in the courts here that Huxmann is not Dean of a reputable college is therefore bought testimony on behalf of the so-called Trust of reputable colleges.

This repetition of a slander, uttered first by Huxmann in Chicago, then by Pitner in a letter to Dr. Crouse of September 1, just before arraignment of Smyser under the evidence elaborated by me in the Finley forgery and the irregular Igney license issue, and later repeated by Huxmann by circulars in Germany, I did not deem worthy of other notice than to report the matter to the State Department for investigation while at Chicago last August (1901) engaged in the elaboration of this material with the Hon. John J. Knickerbocker.

As the State Department has not seen fit to consider the matter further, I am obliged to ask you to certify under oath before a notary public, and have the notary's acknowledgment properly authenticated, for immediate use here in the courts. Your affidavit to embrace:

First. That all moneys contributed by the various bodies were paid over to the investigating committee and none of it to me.

Second. That I was never paid more than entertainment at Milwaukee and Chicago and railroad expenses for trip to the conventions, and never for services rendered the dental profession in my efforts to uncover the frauds perpetrated under the seal of the State of Illinois by certain officials of the Board of Dental Examiners and their punishment for the irregular issue of dental degrees.

Third. That all my expenses for the preparation of my material, my expenditures in photographic reproduction, typewriting, and secretary's services before going West and since my departure from there have been borne by me alone, and that I have never been reimbursed for any such expenditures except while the guest of the convention in Milwaukee and Chicago.

Fourth. That the Committee of Investigation, which handles the funds, has never paid over to me one cent for my services, for my outlays of all kinds, including photographic reproductions, preceding the conventions or since my departure from Chicago, nor any other of my expenditures.

All these expenses have been borne solely by myself, as the government of the United States refused to allow any of these expenditures, even the expense for the reports while at work in the revision and compilation of the material at Washington, as the government holds that it would be a precedent out of harmony with the practices of the State Department. Dr. Truman, in the INTERNATIONAL DENTAL JOURNAL of December, 1901, stated that the expenditures incurred by me should be refunded by the profession, which no doubt feels itself indebted to me not only for initiating this warfare against fraudulent colleges and the dishonest officials of the State of Illinois, but especially for the pains taken by me to cause the establishment of a new and honorable Board in Illinois, the prosecution of Smyser for his forgeries of the minutes in the Finley case, and the illegal issue of the Igney license, all which are the initiative in a work that has since been accomplished by the Committee of Investigation.

Very respectfully yours,

J. H. WORMAN,

*United States Consul-General.*

CONSULATE-GENERAL OF THE UNITED STATES OF AMERICA,
KINGDOM OF BAVARIA, CITY OF MUNICH, GERMAN EMPIRE.

I the undersigned, do hereby certify that the foregoing document is a correct copy of the original on file in this Consulate-General, and as such is entitled to full faith and credit.

Witness my hand and the seal of this Consulate-General, this 19th day of December, 1902.

